# Chronic pain patients can be classified into four groups: Clustering-based discriminant analysis of psychometric data from 4665 patients referred to a multidisciplinary pain centre (a SQRP study)

**DOI:** 10.1371/journal.pone.0192623

**Published:** 2018-02-08

**Authors:** Emmanuel Bäckryd, Elisabeth B. Persson, Annelie Inghilesi Larsson, Marcelo Rivano Fischer, Björn Gerdle

**Affiliations:** 1 Pain and Rehabilitation Centre, and Department of Medical and Health Sciences, Linköping University, Linköping, Sweden; 2 Lund University, Department of Health Sciences, Lund, Sweden; 3 Department of Pain Rehabilitation Medicine, Skåne University Hospital, Lund, Sweden; 4 Quality Stat AB, Umeå, Sweden; Brown University, UNITED STATES

## Abstract

**Objective:**

To subgroup chronic pain patients using psychometric data and regress the variables most responsible for subgroup discrimination.

**Design:**

Cross-sectional, registry-based study.

**Setting and subjects:**

Chronic pain patients assessed at a multidisciplinary pain centre between 2008 and 2015.

**Methods:**

Data from the Swedish quality registry for pain rehabilitation (SQRP) were retrieved and analysed by principal component analysis, hierarchical clustering analysis, and partial least squares–discriminant analysis.

**Results:**

Four subgroups were identified. Group 1 was characterized by low “psychological strain”, the best relative situation concerning pain characteristics (intensity and spreading), the lowest frequency of fibromyalgia, as well as by a slightly older age. Group 2 was characterized by high “psychological strain” and by the most negative situation with respect to pain characteristics (intensity and spreading). Group 3 was characterized by high “social distress”, the longest pain durations, and a statistically higher frequency of females. The frequency of three neuropathic pain conditions was generally lower in this group. Group 4 was characterized by high psychological strain, low “social distress”, and high pain intensity.

**Conclusions:**

The identification of these four clusters of chronic pain patients could be useful for the development of personalized rehabilitation programs. For example, the identification of a subgroup characterized mainly by high perceived “social distress” raises the question of how to best design interventions for such patients. Differentiating between clinically important subgroups and comparing how these subgroups respond to interventions is arguably an important area for further research.

## Introduction

Chronic pain with moderate to severe intensity affects about 20% of the general population [1] and available treatment strategies rarely provide adequate analgesia [2]. A visionary goal for pain medicine would be the ability of basing analgesic treatment and other interventions–e.g., multimodal rehabilitation programs (MMRP)–on a precise understanding of the mechanisms of different pain conditions [3, 4]. It seems probable that prevalent pain syndromes such as chronic widespread pain (CWP) or unspecific chronic low back pain (CLBP) are heterogeneous categories encompassing different (albeit probably inter-related) mechanisms. However, in randomized controlled trials, systematic reviews, and meta-analyses, these broad conditions are often regarded as homogenous categories [5, 6]. Hence, true treatment effects may be “diluted” because only a subgroup of patients respond.

Subgrouping chronic pain patients into different “phenotypes” is therefore an important endeavour. For instance, using quantitative sensory testing, neuropathic pain patients and fibromyalgia patients have been classified into broad phenotypes irrespective of aetiology [7, 8]. Another psychophysical phenotyping method is conditioned pain modulation (CPM) experiments [9]. Subgrouping of pain patients can also be done by combining interview questions with a simple neurological examination [8] or by using self-reported psychometric data from validated questionnaires (e.g., about pain intensity and pain-related interference, quality of life, depression, anxiety, fear-avoidance, acceptance, or catastrophizing) [8, 10, 11]. Hence, it is thought that subgrouping pain patients is an important gateway into a more personalized practice of pain medicine [12–15] and may provide support for health care systems to optimize resources and costs [16]. Identifying subgroups is particularly of interest in conditions such as chronic pain conditions associated with high prevalence, burden, and costs as well as diagnostic and therapeutic uncertainty [17, 18].

Pain is a subjective experience modulated by psychosocial and contextual factors. Engel’s biopsychosocial (BPS) model of disease and [19, 20] has been very influential for pain medicine [21]. Per the BPS model, chronic pain is influenced by and interacts with physical, psychological, and social factors, and pain is often said to have three facets (cognitive-evaluative, sensory-discriminative, and affective-motivational aspect) [22]. It is commonly held that affective factors such as fear and depression, and cognitive factors such as catastrophizing, are important to take into consideration in chronic pain patients. However, the question of causality is not easy to answer [23–26]. The BPS model is also in keeping with the ability of the brain to modulate nociception via top-down pathways [22, 27–29].

Using hierarchical cluster analysis (HCA), this study subgroups patients assessed at a multidisciplinary pain centre using all psychometric data registered in the Swedish quality registry for pain rehabilitation (SQRP) [30]. Based on this cluster analysis, we regressed the variables most responsible for subgroup discrimination to uncover a deeper understanding of what characterised the subgroups. Finally, we investigated whether there was any association between the subgroups and ICD-10 diagnoses.

## Methods

### Subjects and ethics

The participants in this study were patients suffering from chronic pain who were assessed at the Pain and Rehabilitation Centre, University Hospital, Linköping, Sweden between 2008 and 2015 and who were registered in SQRP. The Linköping University Ethics Committee approved the study (Dnr: 2015/108-31); the patients gave their informed written consent in line with the Declaration of Helsinki.

### Psychometric and demografic data

Data were chosen from SQRP. Detailed descriptions of the variables used have been presented elsewhere [31].

#### Demographic data

Age and gender were extracted.

#### ICD codes

ICD-10 codes (string data) were overviewed and, for reasons of consistency, a few five character codes were recoded to four character codes. We focused on diagnoses that were present in ≥1% of all individuals, amounting to 18 diagnoses (including the category “diagnosis missing”), covering 77% of all study subjects.

#### Pain characteristics

Using a numeric rating scale, patients reported last week’s pain intensity (NRS7d). They also denoted the anatomical extent of pain by a pain drawing encompassing 36 anatomical regions; the number of painful regions was thereby registered (NbPainReg; possible range: 0–36). Pain duration in months (PainDur), as well as persistent pain duration (PainDurPer), were also reported by the patients.

#### Hospital anxiety and depression scale (HAD)

HAD assesses anxiety and depression in two subscales of seven item each (HAD-A and HAD-D) [32]. A subscale score of 0–7 is a non-case, 8–10 is a doubtful case, and 11–21 indicates a case. Hence, high subscale scores indicate high levels of depression or anxiety.

#### The West Haven-Yale multidimensional pain inventory (MPI)

MPI consists of three sections. *Part one* has five scales: 1) pain severity (MPI-Sev); 2) pain-related interference in everyday life (MPI-Interf); 3) perceived life control (MPI-Con); 4) affective distress (MPI-Distre); and 5) social support (MPI-Supp). *Part two* has three scales that assess how the pain patient perceives the responses from significant others to expressions of suffering and pain: 1) punishing responses (MPI-Pun); 2) solicitous responses (MPI-Soli); and 3) distracting responses (MPI-Distra). *Part three* has four scales that are synthesized in a composite scale labelled general activity index (MPI-GAI) [33].

#### European quality of life instrument (EQ5D)

This instrument measures how the patient perceives his/her own state of health. EQ5D measures five dimensions: mobility, self-care, usual activities, pain/discomfort, and anxiety/depression. On that basis, an index can be calculated (EQ5D-Index) that ranges from a highest value of 1 (corresponding to best possible health), through 0 (death), to negative values (considered to be “worse than death”) [34]. The EQ5D also measures self-estimated health on a thermometer-like 100-point visual analogue scale (EQ5D-VAS), high values indicating good health.

#### The short form health survey (SF36)

Based on a total of 36 questions, eight different dimensions are assessed on a standardized scale from 0–100: 1) physical functioning, 2) role limitations due to physical functioning, 3) bodily pain, 4) general health, 5) vitality, 6) social functioning, 7) role limitations due to emotional problems, and 8) mental health. High values correspond to high levels of well-being. On that basis, two summary components are calculated, i.e., a physical part (SF36-Phys) and a mental (psychological) part (SF36-Ment) [35].

#### Chronic pain acceptance questionnaire (CPAQ)

The 20-item CPAQ was used [36]. The patient rates each item on a scale from 0 (never true) to 6 (always true), and results are summarized in two subscales: activity engagement (CPAQ-E) with scores ranging from 0–66 and pain willingness (CPAQ-W) with scores ranging from 0–54. High values mirror high activity engagement and high pain willingness, respectively.

#### Tampa scale for kinesiophobia (Tampa)

In this instrument, individuals report fear of movement and (re)injury, i.e., movement is (wrongly) assumed to cause a new injury [37]. A 4-point Likert scale, ranging from ‘‘strongly disagree” to ‘‘strongly agree”, is used on 17 items. Hence, the total score ranges from 17 to 68. The cut-off for kinesiophobia is 36 for women and 38 for men.

#### Life satisfaction questionnaire (LiSat)

This instrument measures patient-reported satisfaction with life as a whole (LiSat-Life) as well as 10 specific areas: vocation (LiSat-Voc), economy (LiSat-Eco), leisure (LiSat-Leis), contacts (LiSat-Cont), sexual life (LiSat-Sex), activities of daily living (LiSat-ADL), family life (LiSat-Fam), partner relationship (LiSat-Part), physical health (LiSat-Phys), and mental health (LiSat-Ment). Each item is graded from 1 (very dissatisfied) to 6 (very satisfied) [38].

### Statistics

#### Multivariate data analysis

We used SIMCA-P+ version 13.0 (Umetrics AB, Umeå, Sweden) for multivariate data analysis by projection (MVDA). More precisely, we performed principal component analysis (PCA), hierarchical clustering analysis (HCA) and, based on the groups defined by HCA, partial least squares–discriminant analysis (PLS-DA). We have previously in detail described the principles of PCA and PLS-DA [10, 39, 40], and this will not be repeated here. Briefly, PCA is a technique that models the correlation structure of a dataset, and thereby enables the identification of multivariate outliers [41, 42]. After outlier detection with PCA, we applied a bottom-up HCA to the principal component score vectors using the default Ward linkage criterion to identify relevant subgroups of patients. HCA complements PCA in the sense that while PCA identifies distinct clusters in multivariate space, HCA can find subtle clusters. In the resulting dendrogram, clusters were identified and, based on these groups, PLS-DA was performed using group belonging as Y-variables and psychometric data as predictors (X-variables). The PLS-DA model was computed to identify associations between the X-variables and the subgroups. This was visualized on a corresponding loading plot.

#### Traditional statistics

Based on the four groups defined by HCA, traditional inferential statistics (Kruskal Wallis Test, Pearson Chi-Square, Mann-Whitney U Test) were computed using IBM® SPSS® Statistics version 23. Effect sizes by Cohen’s d were computed according to the formula

$$d=\frac{mean\;of\;group\;a-mean\;of\;group\;b}{\sqrt{\left((n_{a}-1)*{SD}_{a}^{2}+(n_{b}-1)*{SD}_{b}^{2})/(n_{a}+n_{b}-2\right)}}$$

where n is the number of individuals in group a or b, and SD is the standard deviation of group a or b [43–45]. The effect size was considered very large for │d│ ≥1.3, large for │d│ = 0.80–1.29, moderate for │d│ = 0.50–0.79, small for│d│ = 0.20–0.49, and insignificant for │d│<0.20. For │d│ = 0.80, the mean of one subgroup is at the 79^th^ percentile of the other group. Corresponding figures for│d│ = 1.50, 2.00, 2.50, and 3.00 are the 93^th^, 98^th^, 99^th^, and 99.9^th^ percentiles, respectively [43–45].

## Results

### Data overview and outlier detection

**Fig 1:**
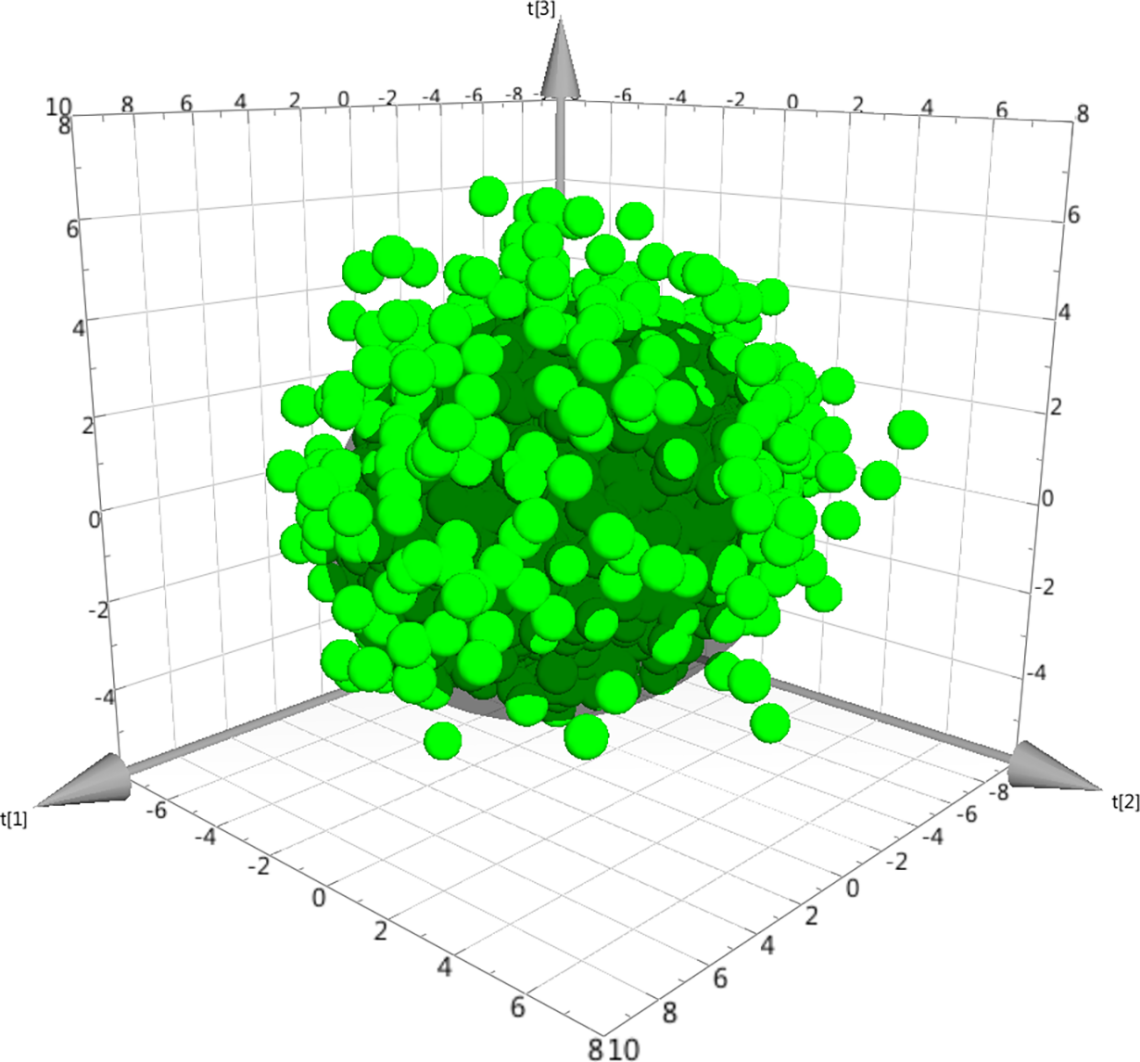
Three-dimensional score plot of the principal component analysis (PCA) model. Each dot represents a patient, and the darker inner sphere represents the Hotelling’s T2 95% confidence interval. As there are >4000 patients, one expects that >200 dots would be outside the sphere. Crucially, the sphere is “well-centred”, i.e., the plot shows that there are no serious outliers left.

Between 2008 and 2015, data were available for 5111 patients. However, 394 patients with >50% missing values were excluded from further analysis. Hence, data from 4717 patients were overviewed by PCA. Because 49 strong outliers and three moderate outliers were excluded, 4665 patients were retained for subsequent analyses. The resulting PCA model (n = 4665, 3 PC, R^2^ = 0.41, Q^2^ = 0.25) had a well-centred score plot (**Fig 1**), in accordance with the removal of strong outliers.

### Hierarchical clustering analysis

**Fig 2:**
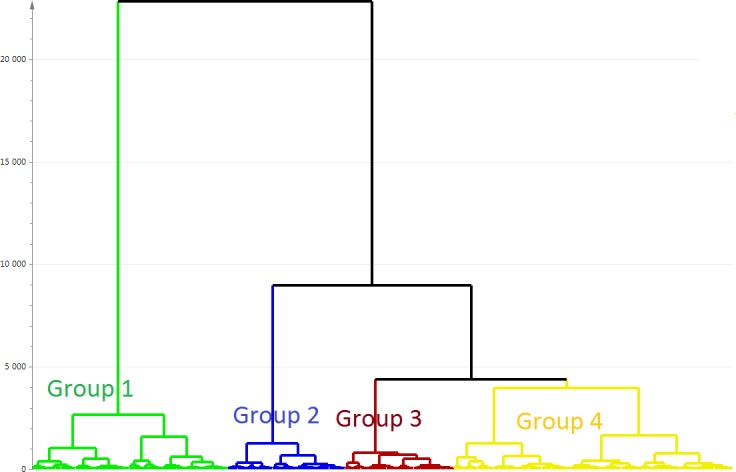
Dendrogram representing four clusters in the hierarchical cluster analysis (HCA). The vertical scale (Y-axis) is a similarity/dissimilarity measure. The individual observations (patients) are on the bottom row (X-axis).

Based on this PCA model, a HCA was performed. In the resulting dendrogram, a level of four clusters/groups was chosen for subsequent analyses (**Fig 2**): group 1 (n = 1305, 28%); group 2 (n = 778, 17%); group 3 (n = 726, 16%); and group 4 (n = 1856, 40%).

### PLS-DA regression

**Fig 3:**
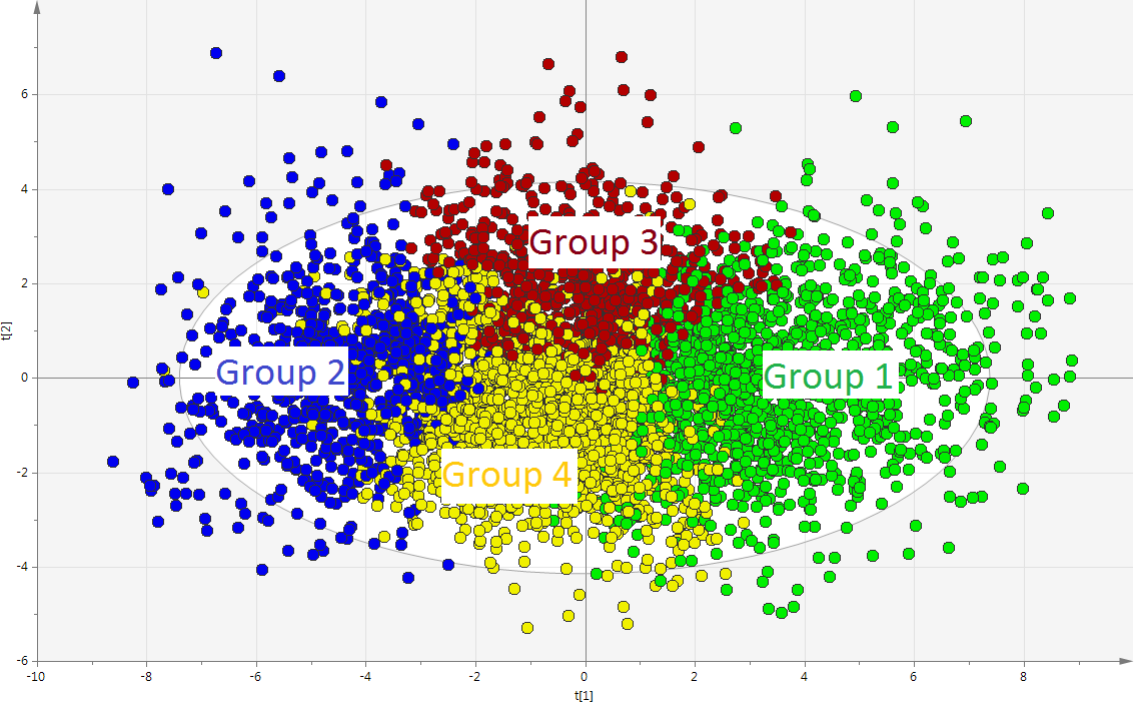
Score plot of the PLS-DA model showing the four clusters. Each dot represents a patient. The two axes–i.e., scores t[1] and t[2]–represent the two latent variables of the model. The latent variables are mathematical constructs that “summarize” the variables registered in the study. PLS-DA: partial least squares–discriminant analysis.

**Fig 4:**
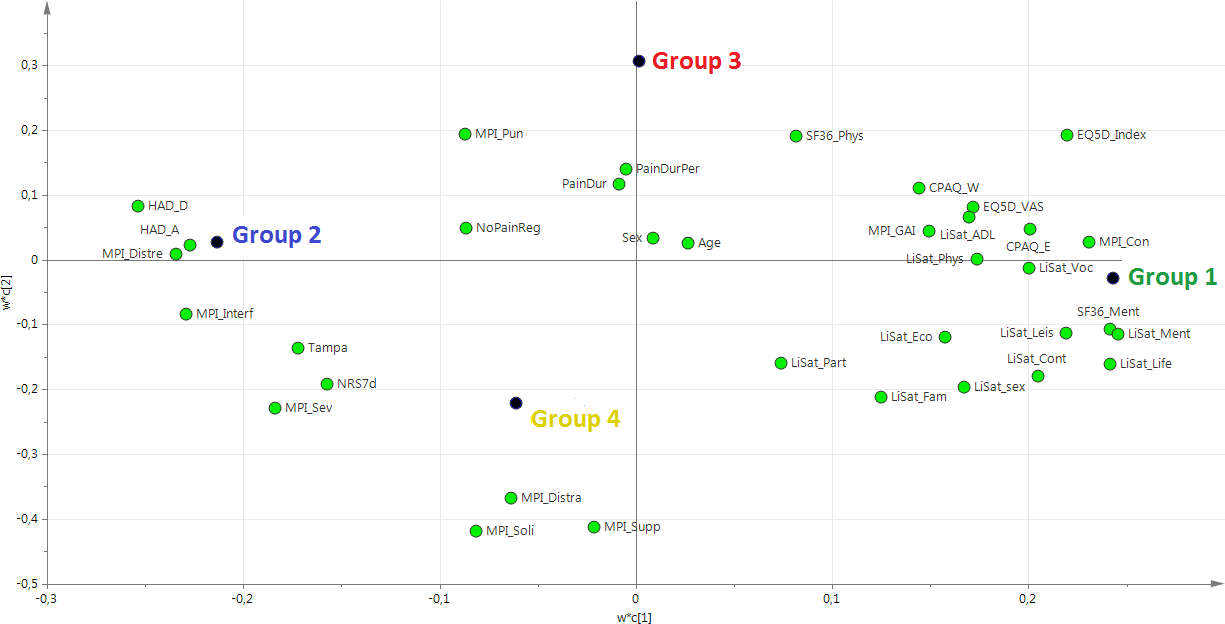
Loading plot of the PLS-DA model. The loading plot is complementary to the score plot and summarizes how the X-variables relate to each other as well as to group belonging (Y-variable symbolized by a group dot). X-variables located near a group dot are positively associated with that group. For instance, group 2 is characterized by high values for HAD_A. Conversely, group 1 is characterized by low values for HAD_A (being located on the opposite side of the origin of the graph). PLS-DA: partial least squares–discriminant analysis.

**Fig 5:**
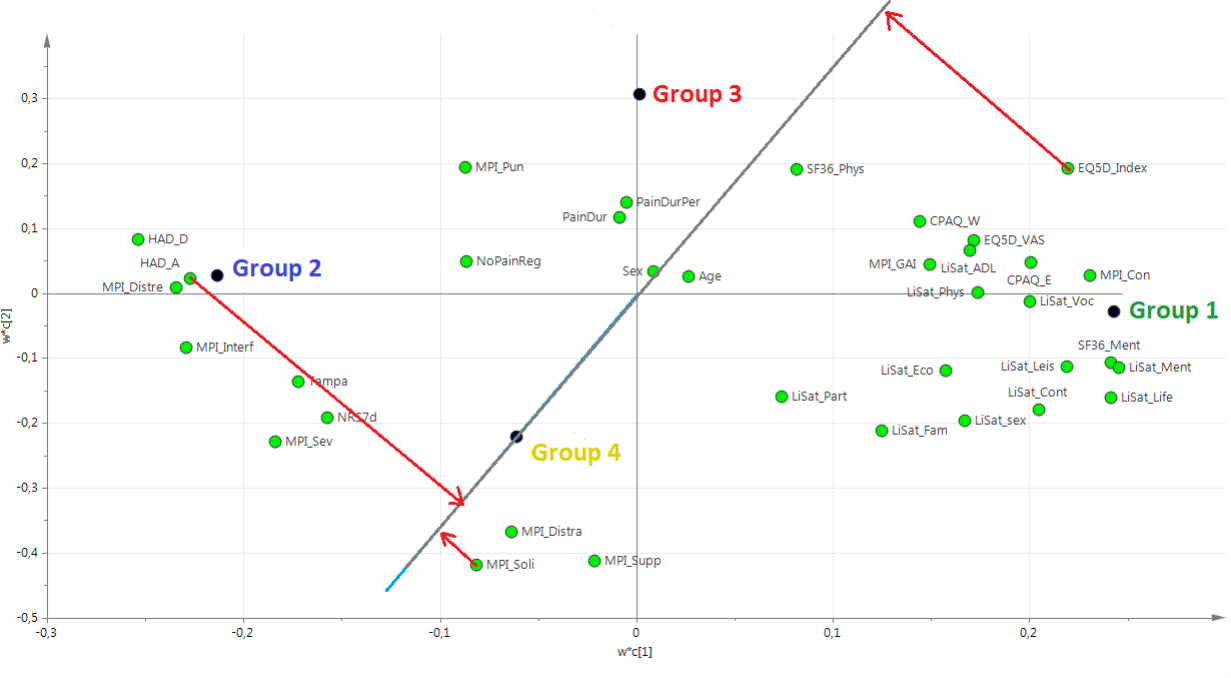
Loading plot of the PLS-DA model, with focus on group 4. To interpret group 4, a new axis passing through the origin and through the group 4 dot has been added. The importance of the X-variables can be assessed by projection of the X-variables on the new axis. For instance, HAD_A and MPI_Soli are rather strongly positively associated with group 4 (i.e., patients in group 4 have high values), whereas EQ5D_Index is strongly negatively associated with group 4 (i.e., patients in group 4 have low EQ5D_Index). PLS-DA: partial least squares–discriminant analysis.

Based on HCA, a PLS-DA model was obtained with group belonging as Y-variable. The model had two latent variables (R^2^ = 0.35, Q^2^ = 0.34, p<0.001 by CV-ANOVA) and the four groups are visualized in **Fig 3**. The corresponding loading plot of the PLS-DA model is shown in **Fig 4**. The interpretation of groups 1, 2, and 3 was simplified by the fact that the “group dot” was almost on either one of the axes of the plot. For group 4, however, this was not the case, and to interpret group 4, an additional interpretative axis was added in the loading plot, in accordance with the standards of how to interpret PLS-DA models (**Fig 5**). The findings of the two loading plots (**Figs 4 and 5**) can be summarized as follows (a selection of variables is mentioned). More detail is provided below.

Group 1 was characterized by the following:

- High quality of life, i.e., positive correlations with LiSat-Life, EQ5D-VAS, and EQ5D-Index.
- Relative good coping and acceptance skills, i.e., positive associations with CPAQ-E and CPAQ-W.
- Good psychological status, i.e., positive correlations with SF36-Ment and LiSat-Ment and negative correlations with HAD-A, HAD-D, and MPi-Distre.
- Low pain intensity (i.e., negative correlations with NRS7d and MPI-severe).

Group 2 was characterized as the opposite of group 1.

Group 3 was characterized by the following:

- High levels of “social distress”, i.e., group 3 correlates positively with MPI-Pun and negatively with MPI-Soli, MPI-Supp, and MPI-Distra.
- Long pain durations (i.e., positive correlations with PainDur and PainDurPer).

Group 4 was in many ways like group 2 in the sense that it shared much of group 2’s characteristics concerning, e.g., HAD-A and the EQ5D-index (**Fig 5**). Moreover, in contrast to group 3, group 4 was characterized by low levels of “social distress”, i.e., group 4 correlated positively with MPI-Soli, MPI-Supp, and MPI-Distra.

### Traditional inferential statistics

In **Table 1**, the four groups are compared by traditional omnibus statistical testing. For post hoc testing, see **Table 2** for p-values and **Table 3** for effect sizes by Cohen’s d. Overall, we found highly significant p-values for all X-variables and |d|≥0.8 (i.e., a large or very large effect size) in 103 out of 204 computations (i.e., in ≈50% of cells in **Table 3**), thereby confirming the validity of the clustering-based PLS-DA model.

**Table 1:** Characteristics of the four clusters.

Variable	Group 1 (n = 1305, 28%)	Group 2 (n = 778, 17%)	Group 3 (n = 726, 16%)	Group 4 (n = 1856, 40%)	Statistics
Gender (% females)	69%	66%	73%	68%	P = 0.032
Age	49 (38–60)	46 (36–55)	46 (37–55)	45 (33–56)	P <0.001
PainDur	1312 (451–3769)	2002 (720–4346)	2800 (870–5910)	1333 (460–3889)	P <0.001
PainDurPer	1018 (377–2937)	1432 (491–3388)	2336 (822–4872)	971 (338–2762)	P <0.001
NRS7d	6 (5–7)	8 (8–9)	7 (5–7)	8 (7–9)	P <0.001
NbPainReg	8 (4–14)	15 (8–24)	13 (7–20)	12 (6–20)	P <0.001
HAD-A	4 (2–6)	14 (11–17)	9 (6–12)	9 (6–12)	P <0.001
HAD-D	4 (2–6)	14 (11–16)	9 (7–11)	9 (6–11)	P <0.001
MPI-Sev	4.0 (3.3–4.7)	5.3 (5.0–6.0)	4.0 (3.7–4.7)	5.0 (4.3–5.3)	P <0.001
MPI-Interf	3.5 (2.6–4.1)	5.5 (5.2–5.8)	4.3 (3.8–4.8)	4.8 (4.3–5.3)	P <0.001
MPI-Con	3.5 (3.0–4.3)	1.3 (0.8–2.0)	2.8 (2.3–3.3)	2.5 (1.8–3.0)	P <0.001
MPI-Distre	2.0 (1.3–3.0)	5.0 (4.3–5.7)	3.7 (3.0–4.0)	3.7 (3.0–4.3)	P <0.001
MPI-Supp	4.3 (3.3–5.0)	4.7 (3.7–5.7)	3.0 (2.0–4.0)	5.0 (4.3–5.7)	P <0.001
MPI-Pun	1.0 (0.3–1.8)	2.3 (1.3–3.5)	2.3 (1.3–3.3)	1.5 (0.8–2.3)	P <0.001
MPI-Soli	2.7 (1.7–3.7)	3.5 (2.3–4.5)	1.7 (1.0–2.5)	3.7 (2.7–4.5)	P <0.001
MPI-Distra	2.3 (1.5–3.0)	2.8 (2.0–3.7)	1.5 (0.8–2.3)	3.0 (2.3–3.8)	P <0.001
MPI-GAI	2.8 (2.3–3.3)	1.5 (0.9–2.1)	2.4 (1.9–2.9)	2.1 (1.6–2.8)	P <0.001
EQ5D-Index	0.62 (0.16–0.73)	-0.08 (-0.18–0.03)	0.26 (0.09–0.66)	0.09 (-0.01–0.16)	P <0.001
EQ5D-VAS	55 (40–70)	20 (12–30)	40 (30–55)	32 (25–47)	P <0.001
SF36-Phys	30.7 (23.8–37.0)	24.3 (20.7–28.6)	30.4 (25.9–35.9)	25.7 (20.3–31.8)	P <0.001
SF36-Ment	50.2 (42.4–55.9)	21.6 (16.7–27.6)	31.7 (25.2–40.6)	33.5 (27.0–42.5)	P <0.001
CPAQ-E	37 (30–43)	12 (7–19)	28 (21–34)	24 (16–31)	P <0.001
CPAQ-W	27 (21–32)	16 (10–22)	24 (20–29)	20 (15–26)	P <0.001
Tampa	35 (30–40)	49 (41–55)	38 (32–43)	42 (37–48)	P <0.001
LiSat-Life	5 (4–5)	2 (1–2)	3 (3–4)	4 (3–4)	P <0.001
LiSat-Voc	4 (3–5)	1 (1–2)	3 (2–4)	2 (1–4)	P <0.001
LiSat-Eco	5 (4–5)	2 (1–3)	3 (2–4)	4 (2–5)	P <0.001
LiSat-Leis	4 (3–5)	1 (1–2)	3 (2–3)	3 (2–4)	P <0.001
LiSat-Cont	5 (4–6)	2 (2–3)	3 (2–4)	4 (3–5)	P <0.001
LiSat-Sex	4 (3–5)	1 (1–2)	2 (1–3)	3 (2–4)	P <0.001
LiSat-ADL	5 (4–6)	3 (2–4)	5 (4–5)	4 (3–5)	P <0.001
LiSat-Fam	5 (5–6)	4 (3–5)	4 (3–5)	5 (4–6)	P <0.001
LiSat-Partn	6 (5–6)	4 (3–6)	4 (3–6)	5 (5–6)	P <0.001
LiSat-Phys	3 (2–4)	1 (1–2)	2 (2–3)	2 (1–3)	P <0.001
LiSat-Ment	5 (4–5)	2 (1–2)	3 (3–4)	4 (3–4)	P <0.001

Data are expressed as median (25th-75th percentiles), except for gender. Statistics computed by Kruskal Wallis Test, except for gender (Pearson Chi-Square). Posthoc statistics are presented in Table 2.

**Table 2:** Posthoc p-values for psychometric variables.

Variable	Group 1 vs 2	Group 1 vs 3	Group 1 vs 4	Group 2 vs 3	Group 2 vs 4	Group 3 vs 4
Gender	P = 0.123	P = 0.108	P = 0.382	P = 0.005†	P = 0.373	P = 0.016†
Age	*	*	*	P = 0.815	P = 0.103	P = 0.078
PainDur	*	*	P = 0.850	*	*	*
PainDurPer	P = 0.002†	*	P = 0.370	*	*	*
NRS7d	*	P = 0.068	*	*	*	*
NbPainReg	*	*	*	*	*	P = 0.066
HAD-A	*	*	*	*	*	P = 0.020†
HAD-D	*	*	*	*	*	P = 0.002†
MPI-Sev	*	P = 0.015†	*	*	*	*
MPI-Interf	*	*	*	*	*	*
MPI-Con	*	*	*	*	*	*
MPI-Distre	*	*	*	*	*	*
MPI-Supp	*	*	*	*	*	*
MPI-Pun	*	*	*	P = 0.427	*	*
MPI-Soli	*	*	*	*	P = 0.036	*
MPI-Distra	*	*	*	*	*	*
MPI-GAI	*	*	*	*	*	*
EQ5D-Index	*	*	*	*	*	*
EQ5D-VAS	*	*	*	*	*	*
SF36-Phys	*	P = 0.823	*	*	*	*
SF36-Ment	*	*	*	*	*	P = 0.001†
CPAQ-E	*	*	*	*	*	*
CPAQ-W	*	*	*	*	*	*
Tampa	*	*	*	*	*	*
LiSat-Life	*	*	*	*	*	*
LiSat-Voc	*	*	*	*	*	P = 0.006†
LiSat-Eco	*	*	*	*	*	*
LiSat-Leis	*	*	*	*	*	*
LiSat-Cont	*	*	*	*	*	*
LiSat-Sex	*	*	*	*	*	*
LiSat-ADL	*	*	*	*	*	*
LiSat-Fam	*	*	*	*	*	*
LiSat-Partn	*	*	*	*	*	*
LiSat-Phys	*	*	*	*	*	*
LiSat-Ment	*	*	*	*	*	*

Posthoc statistics by Mann Whitney U Test, except for Gender (Chi-Square). For median (25^th^-75^th^ percentiles) values, see Table 1. A p-value ≤0.05 was considered significant. For purposes of clarity and because of the great number of highly significant comparisons, P<0.001 is denoted simply by *. All other significant comparisons are denoted by †.

**Table 3:** Posthoc pairwise effect sizes by Cohen’s d.

Variable	Group 1 vs 2	Group 1 vs 3	Group 1 vs 4	Group 2 vs 3	Group 2 vs 4	Group 3 vs 4
Age	+0.15	+0.15	+0.21	+0.00	+0.07	+0.07
PainDur	-0.07	-0.38	-0.04	-0.33	+0.02	+0.32
PainDurPer	-0.06	-0.42	-0.01	-0.38	+0.04	+0.39
NRS7d	-1.26	-0.10	-0.82	+1.29	+0.55	-0.70
NbPainReg	-0.76	-0.51	-0.42	+0.25	+0.32	+0.07
HAD-A	-2.79	-1.28	-1.28	+1.32	+1.15	-0.11
HAD-D	-3.34	-1.85	-1.53	+1.38	+1.44	+0.12
MPI-Sev	-1.60	-0.14	-1.09	+1.71	+0.68	-1.04
MPI-Interf	-2.31	-0.90	-1.56	+1.70	+1.06	-0.62
MPI-Con	+2.33	+0.89	+1.15	-1.53	-1.10	+0.32
MPI-Distre	-2.78	-1.31	-1.47	+1.56	+1.21	-0.20
MPI-Supp	-0.18	+0.90	-0.54	+1.00	-0.31	-1.56
MPI-Pun	-0.97	-0.96	-0.34	+0.06	+0.61	+0.56
MPI-Soli	-0.52	+0.74	-0.68	+1.27	-0.12	-1.46
MPI-Distra	-0.37	+0.61	-0.58	+0.96	-0.19	-1.20
MPI-GAI	+1.46	+0.40	+0.70	-1.09	-0.72	+0.32
EQ5D-Index	+2.08	+0.36	+1.26	-1.81	-0.93	+0.89
EQ5D-VAS	+1.63	+0.59	+1.01	-1.17	-0.65	+0.46
SF36-Phys	+0.45	-0.02	+0.54	-0.95	-0.18	+0.62
SF36-Ment	+2.86	+1.60	+1.39	-1.11	-1.20	-0.15
CPAQ-E	+2.24	+0.87	+1.16	-1.49	-0.99	+0.35
CPAQ-W	+1.24	+0.28	+0.79	-1.06	-0.50	+0.54
Tampa	-1.50	-0.32	-0.88	+1.19	+0.61	-0.57
LiSat-Life	+3.07	+1.57	+1.06	-1.34	-1.51	-0.31
LiSat-Voc	+2.02	+0.91	+0.98	-1.03	-0.81	+0.10
LiSat-Eco	+1.52	+0.95	+0.66	-0.50	-0.75	-0.24
LiSat-Leis	+2.34	+1.28	+1.00	-1.07	-1.10	-0.19
LiSat-Cont	+2.27	+1.41	+0.86	-0.78	-1.13	-0.40
LiSat-Sex	+1.73	+1.13	+0.59	-0.56	-0.95	-0.46
LiSat-ADL	+1.66	+0.41	+0.72	-1.23	-0.76	+0.35
LiSat-Fam	+1.26	+0.99	+0.36	-0.30	-0.81	-0.52
LiSat-Partn	+0.71	+0.59	+0.19	-0.12	-0.51	-0.39
LiSat-Phys	+1.61	+0.65	+0.75	-1.13	-0.75	+0.14
LiSat-Ment	+3.15	+1.57	+1.19	-1.32	-1.36	-0.17

Positive Cohen’s d numbers indicate that the first mean value is larger than the second, and negative numbers indicate the opposite. For instance, positive values in the “Group 1 vs 2” column indicates that mean of group 1 > mean of group 2, whereas negative values indicate that mean of group 1 < mean of group 2. In order to obtain Cohen’s d values, the mean differences between groups were standardized by dividing by the pooled standard deviation, see equation in the Methods section. To facilitate the interpretation of the table, the cells have been color-coded as follows. Red cell: │d│ ≥1.3, i.e., very large effect size. Dark grey cell: │d│ = 0.80–1.29, i.e., large effect size. Grey cell: │d│ = 0.50–0.79, i.e., moderate effect size. Light grey cell: │d│ = 0.20–0.49, i.e., small effect size. White cell: │d│<0.20, i.e., insignificant effect size.

**Fig 6:**
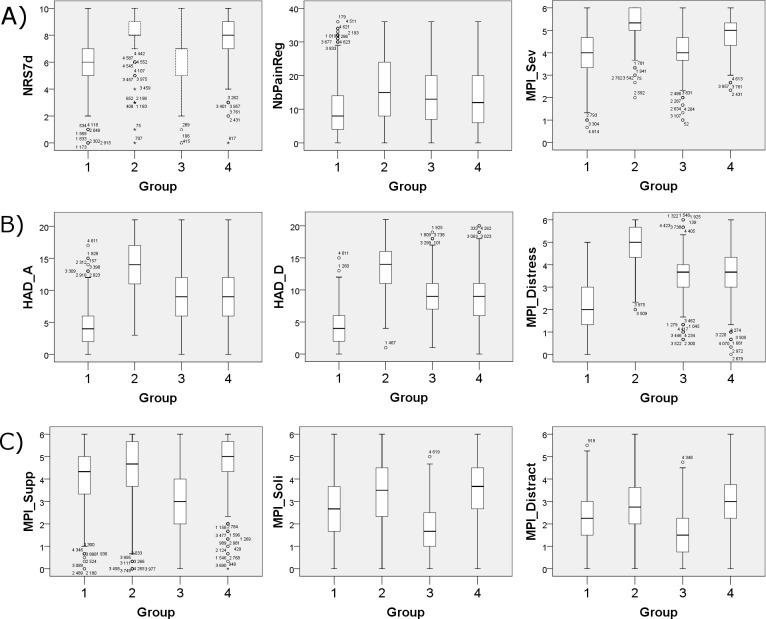
Group differences for 9 selected variables. The boxes represent the interquartile range (IQR) and median values are depicted as horizontal lines in the boxes. The ends of the whiskers depict the lowest and highest datum within 1.5 IQR of the lower or upper quartile, respectively. Points represent outliers and asterisks extremes. For inferential statistics, see Tables 1 and 2. (A) Three directly pain-related variables. From left to right, NRS7d, NbPainReg, and MPI_Sev. (B) Three mental variables. From left to right, HAD_A, HAD_D, and MPI_Distress. (C) Three social variables. From left to right, MPI_Supp, MPI_Soli, and MPI_Distract.

To illustrate important group differences, nine selected variables (three “directly pain-related”, three “psychological”, and three “social”) are displayed as boxplots in **Fig 6** (for significance levels and effect sizes, see **Tables 2** and **3**, respectively).

#### Demographics

Although the model was not influenced very much by sex or age in relation to other X-variables (**Fig 4**), both sex and age were nonetheless statistically significant (**Table 1**).

Sex distribution differed statistically between group 2 and group 3 (P = 0.005) and between group 3 and group 4 (P = 0.016) (**Table 2**). Hence, group 3 was characterized by a higher frequency of females. Although the difference was small (**Table 1**), it is nonetheless notable that females were overrepresented in group 3 and characterized by high “social distress”.

Group 1 was characterized by a statistically higher age than the other groups (**Table 2**). However, the differences were small, i.e., in median 3–4 years.

#### Directly pain-related variables

**Fig 4** suggests that pain intensity (NRS7d and MPI-sev) would be higher in groups 2 and 4, and this was confirmed by traditional statistics (**Tables 1** and **2**).

As seen in **Fig 4**, pain durations (i.e., PainDur and PainDurPer) were positively associated with group 3, which had indeed by far the highest median value of all groups for these variables (**Table 1**).

NbPainReg differed statistically between all groups except between group 3 and 4 (near significance, **Table 2**). Notably, the greatest difference between groups concerning NbPainReg was between groups 1 and 2, the median value being almost twice as high in group 2 (**Table 1**). Hence, spreading of pain was associated with high psychological strain (**Fig 4**).

#### ICD-10 codes

The frequency of the following diagnoses differed between groups: M79.7 fibromyalgia (P<0.001); M54.4 lumbago with sciatica (P = 0.012); G62.9 polyneuropathy, unspecified (P = 0.032); and M79.2 neuralgia, unspecified (P = 0.008) (**Table 4;** post hoc tests in **Table 5**).

**Table 4:** Frequency of ICD-10 codes in the four clusters.

Diagnosis (ICD 10 code)	Group 1 (n = 1305, 28%)	Group 2 (n = 778, 17%)	Group 3 (n = 726, 16%)	Group 4 (n = 1856, 40%)	Statistics (p-value)
Pain, unspecified/Generalized pain NOS (R52.9)	9.5%	12.2%	12.4%	10.5%	0.113
Diagnosis missing in SQPR	10.4%	9.4%	10.7%	10.3%	0.829
Fibromyalgia M79.7	5.7%	12.2%	13.9%	11.0%	<0.001
Lumbago NOS M54.5	8.0%	8.1%	7.0%	9.0%	0.408
Myalgia M79.1	7.7%	6.0%	9.1%	6.8%	0.095
Cervicobrachial syndrome M53.1	5.7%	6.4%	5.9%	5.5%	0.842
Cervicalgia M54.2	3.9%	3.0%	3.0%	3.4%	0.617
Lumbago with sciatica M54.4	3.3%	4.6%	1.7%	3.1%	0.012
Pain in limb M79.6	4.1%	2.7%	2.2%	2.9%	0.085
Sequelae of other specified injuries of neck and trunk T91.8	2.5%	3.6%	3.0%	3.2%	0.460
Cervicocranial syndrome M53.0	1.9%	1.8%	2.1%	2.3%	0.855
Other chronic pain R52.2	1.7%	2.3%	1.7%	2.3%	0.499
Backache NOS M54.9	2.1%	1.5%	1.2%	1.6%	0.511
Pain in thoracic spine M54.6	1.5%	0.8%	1.9%	1.6%	0.282
Cervical disc disorder with radiculopathy M50.1	1.1%	1.3%	1.1%	1.5%	0.782
Polyneuropathy, unspec G62.9	1.5%	1.5%	0.3%	0.9%	0.032
Tension-type headache G44.2	1.5%	0.4%	1.4%	0.9%	0.076
Neuralgia, unspec. M79.2	1.6%	0.4%	0.3%	1.1%	0.008

Diagnosis frequency is expressed as the percentage of diagnoses in that group. Only diagnoses present in ≥1% of all patients have been analysed. Statistics is by Pearson Chi-square. For posthoc testing, see Table 5.

**Table 5:** Posthoc statistics for ICD-10 codes.

Diagnosis	Group 1 vs 2	Group 1 vs 3	Group 1 vs 4	Group 2 vs 3	Group 2 vs 4	Group 3 vs 4
Fibromyalgia M79.7	P<0.001	P<0.001	P<0.001	NS	NS	P = 0.039
Neuralgia, unspec. M79.2	P = 0.011	P = 0.006	NS	NS	NS	P = 0.037
Lumbago with sciatica M54.4	NS	P = 0.029	NS	P = 0.001	P = 0.048	P = 0.045
Polyneuropathy, unspec. G62.9	NS	P = 0.009	NS	P = 0.011	NS	NS‡

Statistics is by Pearson Chi-square. NS: not significant

‡: not statistically significant (p = 0.087) but three times lower frequency in group 3 vs. group 4 (0.3% vs 0.9%).

Fibromyalgia was strongly associated with groups 2, 3, and 4 as opposed to group 1 (**Table 5**); the frequency of fibromyalgia in group 1 was about half the frequency in the other groups (**Table 4**). For fibromyalgia, there was also a statistical association with group 3 in comparison with group 4 (P = 0.039), but the difference was small (13.9% vs. 11.0%).

Moreover, neuropathic pain conditions (i.e., neuralgia, lumbago with sciatica, and polyneuropathy) had low associations with group 3 compared to the other groups (**Tables 4** and **5**).

## Discussion

The major findings of the study were that the four groups/clusters were identified, which had the following characteristics:

- Group 1 was characterized by low psychological strain and the best relative situation with respect to pain characteristics (intensity and spreading) and lowest frequency of fibromyalgia diagnosis as well as a slightly higher age.
- Group 2 was characterized by high psychological strain as well as the most negative situation with respect to pain characteristics.
- Group 3 was characterized by high “social distress”, the longest pain durations, as well as a statistically higher frequency of females. The frequency of three neuropathic pain conditions was generally lower in this group.
- Group 4 was characterized by high psychological strain and low “social distress”, as well as high pain intensity (NRS7d and MPI-sev).

Chronic pain patients are often treated as a homogenous group. Such an indiscriminate approach is problematic [46]. The Swedish Council on Health Technology Assessment (SBU) suggested that RCTs need to describe the included chronic pain patients in a more comprehensive and systematic way [47]. In the present study, four groups were identified. Although significant differences in distribution of certain diagnoses were found, the differences between the groups were small. The importance of the clinical diagnosis seems to be limited when planning for rehabilitation interventions.

Although cognitive behavioural therapy and MMRP are evidence-based treatments [31, 48, 49], only low to medium effect sizes are usually reported. Effect sizes may increase if patients can be classified into meaningful groups [50–52]. Identifying subgroups has been identified as research priority to facilitate development of “tailor-made” interventions [52–54]. Subgrouping of patients with chronic pain has been done in various ways, and there is no consensus concerning the most suitable method and/or data set optimally to be used for subgrouping/clustering [7, 10, 55–58]. From a clinical perspective, it appears important that subgrouping is built on easily assessed and clinically useful data.

Chronic pain is often co-morbid with psychological conditions [59]; 35% of the chronic pain population has co-morbid depression [60] and anxiety is reported in 17 to 35% of chronic pain cohorts [61, 62]. In agreement with earlier studies, these comorbidities were of importance when the four groups were identified especially for groups 1 and 2 [12, 13, 16].

Groups 1 and 2 were the two extremes (together 45% of the cohort) and similar results have been presented earlier [63, 64]. Group 1 presented a relatively good situation concerning pain characteristics, common comorbidities, and health aspects. However, this group still had on average a moderate pain intensity. This group also reported relatively positive situations for psychological status (including satisfaction), kinesiophobia (TAMPA), and acceptance (CPAQ). All subjects included in this study were complex patients in one or several aspects as judged by the refereeing physician (generally in primary health care). Thus, there is a substantial proportion of patents with complex pain conditions who do not report psychological comorbidities such as anxiety and depression [11].

Group 2 was characterized by high psychological strain and high pain intensity. This constellation of psychological symptoms and pain was associated with low health and suffering as demonstrated earlier [14, 64–70]. Patients in group 2 perceived their situation as being “worse than death” (i.e., negative figures) at group level per the EQ5D-Index. The identification of patients belonging to this group seems very important especially since there are reports that such a constellation of symptoms may negatively affect treatment outcomes, feed treatment resistance, and sick leave as well as be associated with high health costs [23, 71–81].

Group 3 was one of two intermediate groups and one of the pregnant characteristics of this group was the perceived lack of social support and support from significant others. Hence, the results of group 3 pinpoint that not only psychological but also social characteristics seem important to consider when assessing chronic pain patients. In a sense, for a subgroup of patients, we have confirmed the importance of the “social” component in the bio-psycho-social model. Further studies on the social dimensions of the chronic pain experience are warranted. Group 3 had a significantly higher proportion of women, and it has recently been shown that women patients (together with patients high in depressive symptoms) seem more vulnerable to spouse criticism/hostility [82]. Social support has been found to be associated with long-term functioning in post neck trauma patients [83]. Future research should evaluate to what extent and how gender aspects contribute to the rating of social support and evaluate whether certain interventions can be implemented to address the low social support, e.g., education for relatives. There is a lack of social support-based interventional studies [84]. As this group was also characterized by long pain durations, it appeared these patients to a certain extent handled their situation without markedly increasing their depressive and anxiety symptoms; this was not the case for group 2, where the highest values were found.

Group 4 –the second intermediary group–was the largest group (40%) of all the four groups and generally (except for two variables) (**Tables 1, 2** and **3**) had a somewhat worse (significant) situation than group 3, although these differences were small. Group 4 also differed significantly from group 2 (except for age and gender). Variables reflecting the perceived health situation (e.g., EQ5D-Index and EQ5D-VAS) were markedly lower in group 4 than in groups 1 and 3 (**Table 1**). Interestingly, group 4 was also characterized by relatively short pain durations compared to groups 2 and 3 (no difference compared to group 1). It seems that patients in group 4 are at risk for developing a clinical picture similar to group 2; however, properly addressing this speculation would require longitudinal studies.

In the future, subgrouping patients into meaningful clusters might have profound clinical implications. Should for instance group 2 focus (at least initially) on pharmacological treatment of depression and/or anxiety, and should group 1 focus on e.g. increased physical activity and not psychological treatment? Is perhaps group 3 in dire need of involving relatives in the treatment programme, for instance through education sessions? And, given that cognitive behavioural therapy (CBT) is less effective in patients with high levels of emotional problems [85], is perhaps group 4 better suited for CBT-based rehabilitation programmes than group 2? All of this is highly speculative but illustrates the potential of using complex patient-related outcomes measures (PROM) in order to hopefully increase the effect size of our interventions. A visionary goal for a truly personalized practice of pain medicine would be to combine PROM with clinical judgement (expressed in e.g. a diagnosis according to the upcoming 11^th^ version of ICD, where chronic pain diagnoses will have their own section) or even perhaps with new biomarkers and/or psychophysical tests mirroring chronic pain pathophysiology [86–89], in order to choose the best treatment for each individual patient.

### Strengths and limitations

One of this study’s strengths is the use of data from a steady influx of patients to a multidisciplinary pain centre responsible for the care of patients with severe chronic pain in a county in Sweden. The large sample size was associated with good power for identifying the four groups. On the other hand, these patients were mainly refereed from the primary care and hence represent a selection of the most complex chronic pain cases. Another strength is that this study was based on a wide array of aspects per the BPS model and in agreement with important facets suggested by ICF and the IMMPACT group. Using MVDA was also a strength since these methods are especially designed to handle and take advantage of the complex intercorrelation pattern of the investigated variables, and using this methodology we found substantial effect sizes (**Table 3**)–confirming the validity of the MVDA methodology and suggesting that the subgroups might be clinically relevant. The cross-sectional study design was unable to identify directions of causality. Another limitation was that we used questionnaires instead of a face-to-face clinical examination of anxiety and depression, which has been shown to be associated with more robust assessments; however, systematic clinical assessments of depression and anxiety were not within the economic resources of the present project. The only systematic clinical assessment was the medical diagnosis.

### Conclusion

Using MVDA four subgroups/clusters were identified. One group (group 2) was characterized by high psychological strain as well as the most negative situation with respect to pain characteristics and health aspects. The other extreme (group 1) had relatively low intensity of symptoms and a relatively good health situation. Two intermediary groups were also found; one of these (group 3) was characterized by high “social distress”, the longest pain durations, as well as a statistically higher frequency of females. The fourth group (group 4) had a somewhat worse situation than group 3 but was also associated with relatively short pain durations. One can speculate that patients in group 4 could be at risk for developing a clinical picture similar to group 2. The four groups showed marked differences in clinical pictures. This may indicate the need to design “tailor-made” interventions.
